# Development, Validation and Clinical Evaluation of a Low Cost In-House HIV-1 Drug Resistance Genotyping Assay for Indian Patients

**DOI:** 10.1371/journal.pone.0105790

**Published:** 2014-08-26

**Authors:** Arpan Acharya, Salil Vaniawala, Parth Shah, Rabindra Nath Misra, Minal Wani, Pratap N. Mukhopadhyaya

**Affiliations:** 1 Department of Molecular Biology, Dr. D. Y. Patil Biotechnology & Bioinformatics Institute, Dr. D. Y. Patil Vidyapeeth, Pune, Maharashtra, India; 2 Department of Molecular Diagnostics, SN Genelab, Surat, Gujarat, India; 3 Department of Molecular Diagnostics, Supratech Micropath Laboratory & Research Institute, Ahmedabad, Gujarat, India; 4 Department of Microbiology, Padmashree Dr. D. Y. Patil Medical College & Research Centre, Dr. D. Y. Patil Vidyapeeth, Pune, Maharashtra, India; 5 Department of Microbiology, Interdisciplinary Science, Technology and Research Academy, AISC, Pune, Maharashtra, India; Alberta Provincial Laboratory for Public Health, University of Alberta, Canada

## Abstract

Human Immunodeficiency Virus-1 (HIV-1) drug resistance genotyping assay is a part of clinical management of HIV-1 positive individuals under treatment with highly active antiretroviral therapy (HAART). Routine monitoring of drug resistance mutations in resource limited settings like India is not possible due to high cost of commercial drug resistance assays. In this study we developed an in-house, cost effective HIV-1 drug resistance genotyping assay for Indian patients and validated it against the US-FDA-approved ViroSeq HIV-1 drug resistance testing system. A reference panel of 20 clinical samples was used to develop and validate the assay against ViroSeq HIV-1 drug resistance testing system which was subsequently used to genotype a clinical panel of 225 samples. The Stanford HIV database was used to identify drug resistant mutations. The analytical sensitivity of the assay was 1000 HIV-1 RNA copies/ml of plasma sample while precision and reproducibility was 99.68±0.16% and 99.76±0.18% respectively. One hundred and one drug resistant mutations were detected by the in-house assay compared to 104 by ViroSeq system in the reference panel. The assay had 91.55% success rate in genotyping the clinical panel samples and was able to detect drug resistant mutations related to nucleoside reverse transcriptase inhibitor (NRTI), non-nucleoside reverse-transcriptase inhibitor (NNRTI) as well as protease inhibitor (PI) classes of antiretroviral drugs. It was found to be around 71.9% more cost effective compared to ViroSeq genotyping system. This evaluation of the assay on the clinical panel demonstrates its potential for monitoring clinical HIV-1 drug resistance mutations and population-based surveillance in resource limited settings like India.

## Introduction

Introduction of antiretroviral therapy (ART) all over the world has dramatically improved the health condition of HIV-1 infected individuals [1]. This is reflected by way of sharp fall in mortality and morbidity associated with HIV-1/AIDS [2]. Developing countries like India with heavy burden of HIV-1 population has also seen a gradual increase in use of ART and at present 0.6 million individuals are taking 1^st^ line ART [3]. In order to sustain the success of this ART program it is important to monitor the patients in regular interval and keep switching the treatment regimen [4] as per need of the patient. As HIV-1 drug resistance is one of the formidable causes for treatment failure and choice for drugs are limited, there is need for a cost effective HIV-1 drug resistance monitoring system in resource limited settings like India [5].

In order to monitor progression of disease in patients undergoing ART, a number of tools such as point-of-care CD4 testing, HIV-1 viral load testing and HIV-1 drug resistance analysis using plasma/dried blood spots are being evaluated for their suitability in resource limited setting in terms of cost and efficiency [6]–[9]. Among these, HIV-1 drug resistance genotyping assay is of special relevance because of its use in monitoring transmitted drug resistance mutations in drug naïve individuals [10]. Information regarding emergence and prevalence of HIV-1 drug resistance mutations is important in guiding the national strategy for implementing ART program in resource limited settings with limited treatment options [11].

The commercial kits available for HIV-1 drug resistance genotyping are expensive and the prohibitive running costs hinder their routine use in resource limited countries like India [12]. These assays are designed specifically for HIV-1 subtype B strains predominant in North America and Europe. Hence, genetic diversity of non B subtype HIV-1 strains prevalent in Africa and Asia pacific region poses challenge for use of these kits in such geographical regions [13]. This has led to continuous efforts to develop homebrew HIV-1 drug resistance genotyping assays across the world with varying success [14]–[16]. India is dominated by HIV-1 subtype C virus [17]. Recently we have demonstrated a definite geographical bias of nucleotide sequence motifs within specific regions of HIV-1 genome [18] that indicate the need for not only cost effective but also a region-specific, high quality HIV-1 drug resistance assay for periodic monitoring of patients undergoing ART.

In this study we report a cost effective HIV-1 drug resistance genotyping assay for Indian population that is validated against US-FDA approved ViroSeq Genotyping System 2.0 (Celera Diagnostics, USA) and demonstrate its efficiency in identifying all drug resistant mutations related to NRTI, NNRTI and PI classes of antiretroviral drugs.

## Materials and Methods

### Study Population

Plasma samples collected form 20 treatment experienced HIV-1 positive patients from India and those experiencing virologic failure were used as reference panel in this study. The clinical characteristics of this resource population are described in **Table 1**.

**Table 1:** Clinical characteristics and subtype of the reference panel.

Sample ID	Plasma Viral Load(HIV-1 RNA copies/ml)	ART Regimen	HIV-1subtype	Plasmagenotype	
				ViroSeq	In-house
HIV-IN-638	1680600	AZT+3TC+EFV	C	+	+
HIV-IN-642	2576440	AZT+3TC+NVP	C	+	+
HIV-IN-648	6598655	AZT+3TC+EFV	C	+	+
HIV-IN-652	1154880	TDF+3TC+NVP	C	+	+
HIV-IN-655	2237655	ATV/r+TDF+3TC	C	+	+
HIV-IN-661	875600	TDF+3TC+EFV	C	+	+
HIV-IN-662	165895	AZT+3TC+NVP	C	+	+
HIV-IN-665	436278	TDF+3TC+NVP	C	+	+
HIV-IN-669	278354	AZT+3TC+EFV	C	+	+
HIV-IN-670	122400	AZT+3TC+EFV	C	+	+
HIV-IN-672	24388	TDF+3TC+EFV	C	+	+
HIV-IN-675	46544	ATV/r+TDF+3TC	C	+	+
HIV-IN-678	75390	TDF+3TC+EFV	C	+	+
HIV-IN-679	33175	TDF+3TC+NVP	C	+	+
HIV-IN-683	12540	AZT+3TC+NVP	A	+	+
HIV-IN-685	8755	AZT+3TC+NVP	C	+	+
HIV-IN-688	4655	ATV/r+TDF+3TC	C	+	+
HIV-IN-690	2690	AZT+3TC+EFV	C	+	+
HIV-IN-694	2100	AZT+3TC+EFV	A	+	+
HIV-IN-695	1350	AZT+3TC+NVP	C	+	+

“+”: Positive amplification; ART: Anti retroviral therapy; AZT: Zidovudine; 3TC: Lamivudine; EFV: Efavirenz; NVP: Nevirapine; TDF: Tenofovir; ATV/r: Atazanavir/r.

The clinical panel used in this study comprised of 225 samples. The patients were enrolled during the period of May 2012 and Septembers 2013 from India and all were under first-line of antiretroviral therapy for more than 6 months and failing the highly active anti retroviral therapy [HAART] as per current HIV treatment guidelines followed in India [19]. After developing the in-house genotyping assay described here, this clinical panel was used to study the prevalence of HIV-1 drug resistance mutations in India. The details of demographic and clinical characteristics of these 225 samples are summarized in **Table 2**. The study was duly approved by SN Gene laboratory clinical research, institutional bio-safety and bio-ethics committee (Approval number DGL/2012/WY07). Written informed consent was obtained from all participants enrolled in this program.

**Table 2:** Demographic characteristics and laboratory results of the clinical panel.

Variables	Summary, n = 225
Age (yrs), median (IQR)	32 (26–41)
Gender, n (%):	
Male	121 (53.7%)
Female	90 (40.0%)
Child	14 (6.3%)
Median CD4T cellcount, cells/µl (IQR)	137 (100–180)
Median Viral load, log10copies/ml (IQR)	5.055 (4.39–5.47)
Risk exposure, n (%):	
Heterosexual (%)	115 (51.2%)
Bisexual (%)	40 (17.7%)
MSM (%)	50 (22.2%)
MTC (%)	20 (8.9%)
Other co-infections, n (%)	48 (21.3%)
HIV-1 subtypes, n (%):	
Subtype C	176 (85.5%)
Subtype A	29 (14.0%)
Subtype B	1 (0.5%)
Treatment regimen:	
AZT, 3TC, EFV	35 (15.56%)
AZT, 3TC, NVP	50 (22.22%)
TDF, 3TC, NVP	45 (20.00%)
TDF, 3TC, EFV	35 (15.56%)
ATV/r, TDF, 3TC	35 (15.56%)
LPV/r, AZT, 3TC	25 (11.10%)

IQR: Interquartile range; MSM: Men who have Sex with Men; MTC: Mother to Child Transmission; AZT: Zidovudine; 3TC: Lamivudine; EFV: Efavirenz; NVP: Nevirapine; ATV/r: Atazanavir/r; LPV/r: Lopinavir/r.

### Specimen Collection and storage

Ten ml of blood was collected from each panel members in K_2_-EDTA vacutainer tubes (Becton Dickinson, San Diego, California, USA). Out of this, 2–3 ml was used for CD4+ T cell counting and the remaining for plasma separation. Plasma samples were stored in 1 ml aliquots at −20°C till further use.

### HIV-1 Viral Load and CD4+ T cell count estimation

The viral load of reference and clinical panel samples were determined using *artus* HIV-1 RG RT-PCR kit (Qiagen, Germany) while CD4/CD8+ T cell counts were estimated using a FACS CALIBUR flow cytometer (BD Biosciences, California, USA), both according to respective manufacturer’s instructions.

### ViroSeq genotyping system

The HIV-1 drug resistance genotyping of reference panel samples were carried out using US-FDA approved ViroSeq genotyping system according to manufacturer’s instructions. ViroSeq HIV-1 genotyping system software v2.6 and Stanford HIVDB [20] were used for drug resistance interpretation.

### RNA Extraction and In-house HIV-1 Drug Resistance Genotyping

The drug resistance genotyping analysis of reference as well as clinical panel samples were carried out according to the method described as follows:

HIV-1 RNA was extracted from plasma samples stored at −20°C within 7 days of collection using a QIAamp Viral RNA mini kit (Qiagen, Germany) and subjected to one step RT PCR. This was followed by nested PCR to generate a 1614 bp amplicon that covered the entire protease gene and more than 300 initial amino acids of reverse transcriptase gene. All primers described in this study were designed using HIV-1 *pol* gene sequences reported from India and available from NCBI GenBank.

Briefly, the extracted RNA samples were reverse transcribed and then amplified using SuperScript III One-Step RT-PCR System (Life Technologies, Foster City, USA). Fifty µl of a reaction mixture comprised of 25 µl of 2X reaction mix, 2 µl of enzyme mix (SuperScript III RT and Platinum Taq), 20 µl of RNA and each primers (forward and reverse) at a final concentration of 10 pmoles per reaction respectively. The primer sequences used in the reaction were: 5′- GCTGTTGGAAATGTGGAA–3′ (forward) and 5′- TGGCTTGCCAATAGTCTGT–3′ (reverse). The thermal cycling profile comprised of 60 minutes of reverse transcription at 45°C followed by 5 minutes of heating at 95°C and 35 subsequent cycles of PCR amplification, each comprising of 95°C for 30 seconds, 56°C for 45 seconds, and 72°C for 180 seconds followed by a 10 minutes final extension at 72°C.

For nested PCR, 5 µl of the 1^st^ round PCR product was used as template. The reaction mixture comprised of 25 µl 2X PCR Mix v.2.0 (TaKaRa-bio, CA, USA), 10 pmoles of each primer (forward and reverse) and nuclease free water to make the volume to 50 µl. The thermal cycling profile comprised of an initial denaturation step of 5 minutes at 95°C followed by 35 cycles of PCR amplification, each comprising of 95°C for 30 seconds, 59°C for 45 seconds, and 72°C for 180 seconds followed by 10 minutes of final extension at 72°C. The primer sequences used for the nested PCR reaction were 5′- GTGGAAAGGAAGGACACCA–3′ (forward) and 5′- TGTTTTACATCATTAGTGT–3′ (reverse). PCR products generated from nested PCR were run on a 1% agarose gel (Promega Corporation, Madison, USA), stained with ethidium bromide (0.5 µg/ml), visualized under a UV source (260 nm) and documented using an automated gel documentation system (BIORAD, USA). For quality control, negative, low positive and high positive control samples were run with every batch of reactions.

PCR products generated from nested PCR were purified using a PureLink Quick PCR Purification Kit (Invitrogen, USA) and subjected to double strand DNA sequencing using 4 pairs of sequencing primers. The sequencing reactions were carried out using the BigDye Terminator v3.1 Cycle Sequencing Kit (Applied Biosystems, USA) as per manufacturer’s instructions followed by capillary electrophoresis performed on an ABI PRISM 3500 Dx Genetic Analyzer (Applied Biosystems, USA). The nucleotide sequences of all oligonucleotide primers used to generate bidirectional sequence data, apart from those used for nested PCR amplification, were as follows: 5′- GTACAGTATTAGTAGGAC–3′, 5′- ATATCAATATAATGTGC–3′, 5′- ATGATATACAGAAGTTAGT–3′, 5′- TACTGGTACAGTTTCAATA–3′, 5′- TGTTTATACTAGGTATGGT–3′ and 5′- CTGGCAGCTGTATAGGCTGTA–3′.

The raw nucleotide sequence data generated were manually edited and assembled into a single contiguous sequence, archived and compared with standard HIV-1 reference strain sequence (HXB2) to obtain the nucleotide variation data. For determining HIV-1 subtype and obtaining HIV-1 drug resistance mutation profile, the edited nucleotide sequences were analyzed using Stanford HIVDB [20]. Single letter amino acid codes were used throughout the manuscript as per the standard IUPAC nomenclature.

### Validation criteria

Validation of the assay methodology described in this study was performed according to WHO guidelines [21].

### Accuracy

Accuracy of the assay was evaluated by analyzing the degree of concordance between drug resistance mutations identified by ViroSeq genotyping system and in-house assay using the reference panel as per IAS mutation list [22].

### Sensitivity

The sensitivity of the assay was evaluated using 5 clinical samples taken from the reference panel. A dilution series was prepared for each sample with viral load of 100000, 10000, 5000, 1000 and 500 HIV-1 RNA copies/ml and tested in triplicate using the in-house assay protocol.

### Precision and Reproducibility

Precision and reproducibility of the assay were evaluated using five clinical samples taken from the reference panel and tested in five replicates each. The degree of concordance of drug resistance associated mutations and nucleotide sequence identity was used to estimate the precision and reproducibility.

### Phylogenetic analysis

Phylogenetic tree was constructed using Neighbor-Joining method [23] in MEGA 5.1 software [24] where the percentage of replicate trees in which the associated taxa clustered together in the bootstrap test (1000 replicates) are shown above the branches [25]. The evolutionary distances were computed using the Maximum Composite Likelihood method and are in units of number of base substitutions per site. The rate variation among sites was modeled with a gamma distribution (shape parameter = 1).

The reference panel tree had 129 sequences, which included 20 reference panel samples each tested with in-house genotyping assay (n = 20) and ViroSeq genotyping system (n = 20) respectively, 50 sequence data generated from 5 clinical samples, each tested in 5 replicates for precision data (n = 25) and reproducibility data (n = 25) respectively and 39 HIV-1 group M reference sequences obtained from HIV sequence database (http://www. hiv. lanl. gov/content/index) maintained by the Los Alamos National Laboratory, University of California, USA. The clinical panel tree comprised of 245 sequences which included 39 HIV-1 subtype reference sequences as described above and 206 clinical panel sequences.

### Statistical Analysis

The clinical and biological parameter of study subjects in reference and clinical panels are presented in frequency (%) for categorical variables. For quantitative variables, data are presented in mean *±* standard deviation (SD) or median [Interquartile range (IQR)]. Prevalence of drug resistance mutations were computed with 95% confidence interval (CI).

#### Nucleotide sequence accession numbers

The GenBank accession number of the sequences generated in this study is KJ185171–KJ185376.

## Results

### Assay design

The present assay is optimized on a nested RT-PCR based protocol to achieve maximum possible sensitivity. The PCR amplicon covered entire protease gene and 1^st^ 300 amino acids of RT gene so as to include all major drug resistance mutations as per the IAS mutation list. The primers used in the study were designed using a database of HIV-1 pol gene sequences reported from India and archived at NCBI GenBank, USA.

### Accuracy

The 20 reference panel samples were genotyped using the ViroSeq genotyping system as well as the in-house genotyping assay. The mean nucleotide and amino acid identity between the two tests were 99.21±0.58% and 99.65±0.43% respectively. A total 101 drug resistance mutations were detected by the in-house assay compared to 104 using the ViroSeq genotyping system. A comparative analysis of drug resistance mutations detected by both the methods is described in **Table 3**.

**Table 3:** Comparison of drug resistance mutations identified by ViroSeq gentyping system and the in-house assay.

Sample ID	Mutations			
	Protease gene		RT gene	
	ViroSeq	In-house	ViroSeq	In-house
HIV-IN-638	None	None	K103N, M184V,Y188L	K103N,M184V, Y188L
HIV-IN-642	None	None	None	None
HIV-IN-648	M46V, I54V,V82A	M46V, I54V, V82A	K103N, M184V	K103N, M184V
HIV-IN-652	L10F, I54V,V82F	L10F, I54V,V82F	T215I	T215I
HIV-IN-655	**L10I**	None	M184V	M184V
HIV-IN-661	A71T	A71T	K103N, V108I,M184V	K103N,V108I, M184V
HIV-IN-662	L10I, L24I, K43T,M46I, I54V,A71V, V82A	L10I, L24I, K43T,M46I, I54V,A71V, V82A	A62V, V75I, F77L, Q151M	A62V, V75I, F77L, Q151M
HIV-IN-665	L10V, G48V,**F53L**, I54V,V82A	L10V, G48V,I54V, V82A	K103N, T215Y	K103N, T215Y
HIV-IN-669	None	None	M41L, M184V,Y188L, T215Y	M41L,M184V, Y188L,T215Y
HIV-IN-670	A71T	A71T	A98G, M184V,G190A, P236L	A98G, M184V,G190A, P236L
HIV-IN-672	L10F	L10F	K65R, K103N,Y181C, M184V	K65R, K103N,Y181C, M184V
HIV-IN-675	I54V, A71V, N88D, L90M	I54V, A71V,N88D, L90M	K70R	K70R
HIV-IN-678	None	None	K65R, K103N,M184V, Y188L,M230L	K65R, K103N,M184V, Y188L, M230L
HIV-IN-679	None	None	M41L, K103N,M184V, T215Y,P225H	M41L, K103N,M184V, T215Y, P225H
HIV-IN-683	L10I	L10I	M41L, D67N,K70R, L74I, A98G,K103N, M184V,T215F, K219Q,P225H	M41L, D67N,K70R, L74I, A98G, K103N, M184V, T215F, K219Q, P225H
HIV-IN-685	**L10I**, M46I,I50L, Q58E,V82C, L90M	M46I, I50L,Q58E, V82C,L90M	M41L, D67N,V75M, K103N,M184V, L210W,T215Y	M41L, D67N,V75M, K103N, M184V, L210W, T215Y
HIV-IN-688	None	None	V106M, G190A	V106M, G190A
HIV-IN-690	G48V, I54V,V82A	G48V, I54V,V82A	A98G, F116Y,Q151M, Y181C,M184V, G190A	A98G, F116Y,Q151M, Y181C, M184V, G190A
HIV-IN-694	None	None	None	None
HIV-IN-695	I54V, V82A	I54V, V82A	Y188L, H221Y	Y188L, H221Y

The discordant mutations are shown in bold and underlined letters.

### Sensitivity

The assay was optimized for amplification of plasma samples having 1000 HIV-1 RNA copies/ml and above. This limit of detection was established by testing a dilution series of 5 samples in triplicates. The assay result is described in **Table 4**. It was found that up to 1000 HIV-1 RNA copies/ml, all replicates of 5 clinical samples amplified successfully but at 500 HIV-1 RNA copies/ml the results were inconsistent.

**Table 4:** Assay sensitivity results using a dilution series from 5 reference panel samples, each tested in triplicate.

Sample ID	Sensitivity at a dilution (HIV-1 RNA copies/ml) of:				
	100000	10000	5000	1000	500
HIV-IN-652	+ + +	+ + +	+ + +	+ + +	− + +
HIV-IN-655	+ + +	+ + +	+ + +	+ + +	+ + −
HIV-IN-665	+ + +	+ + +	+ + +	+ + +	− + −
HIV-IN-669	+ + +	+ + +	+ + +	+ + +	− − −
HIV-IN-670	+ + +	+ + +	+ + +	+ + +	+ − −

“+”: Positive amplification; “–”: Negative amplification.

### Precision and Reproducibility

All five replicates of 5 clinical samples could be amplified and sequenced successfully. The mean nucleotide sequence identity for precision varied between 99.68±0.16% and 100% whereas the mean nucleotide sequence identity for reproducibility varied between 99.76±0.18% and 100%. The results are summarized in **Table 5**. No discordant drug resistance mutations were detected in the replicate data generated for precision and reproducibility.

**Table 5:** Reproducibility and precision data of the in-house assay.

Sample ID	Plasma ViralLoad (HIV-1RNA copies/ml)	HIV-1Subtype	% Nucleotidesequence identity	Replicate Tests						
				Number of drugresistance mutations					Number ofdiscordant mutations	
				A	B	C	D	E		
Reproducibility:										
HIV-IN-638	1680600	C	100.00±0.00%	3	3	3	3	3		0
HIV-IN-661	875600	C	99.81±0.15%	4	4	4	4	4		0
HIV-IN-662	165895	C	99.96±0.07%	11	11	11	11	11		0
HIV-IN-694	2100	A	99.76±0.18%	0	0	0	0	0		0
HIV-IN-695	1350	C	99.84±0.09%	4	4	4	4	4		0
Precision:										
HIV-IN-642	2576440	C	99.68±0.16%	0	0	0	0	0		0
HIV-IN-652	1154880	C	99.89±0.08%	4	4	4	4	4		0
HIV-IN-675	46544	C	99.70±0.21%	5	5	5	5	5		0
HIV-IN-683	12540	A	100.00±0.00%	11	11	11	11	11		0
HIV-IN-690	2690	C	99.93±0.07%	9	9	9	9	9		0

**Figure 1:**
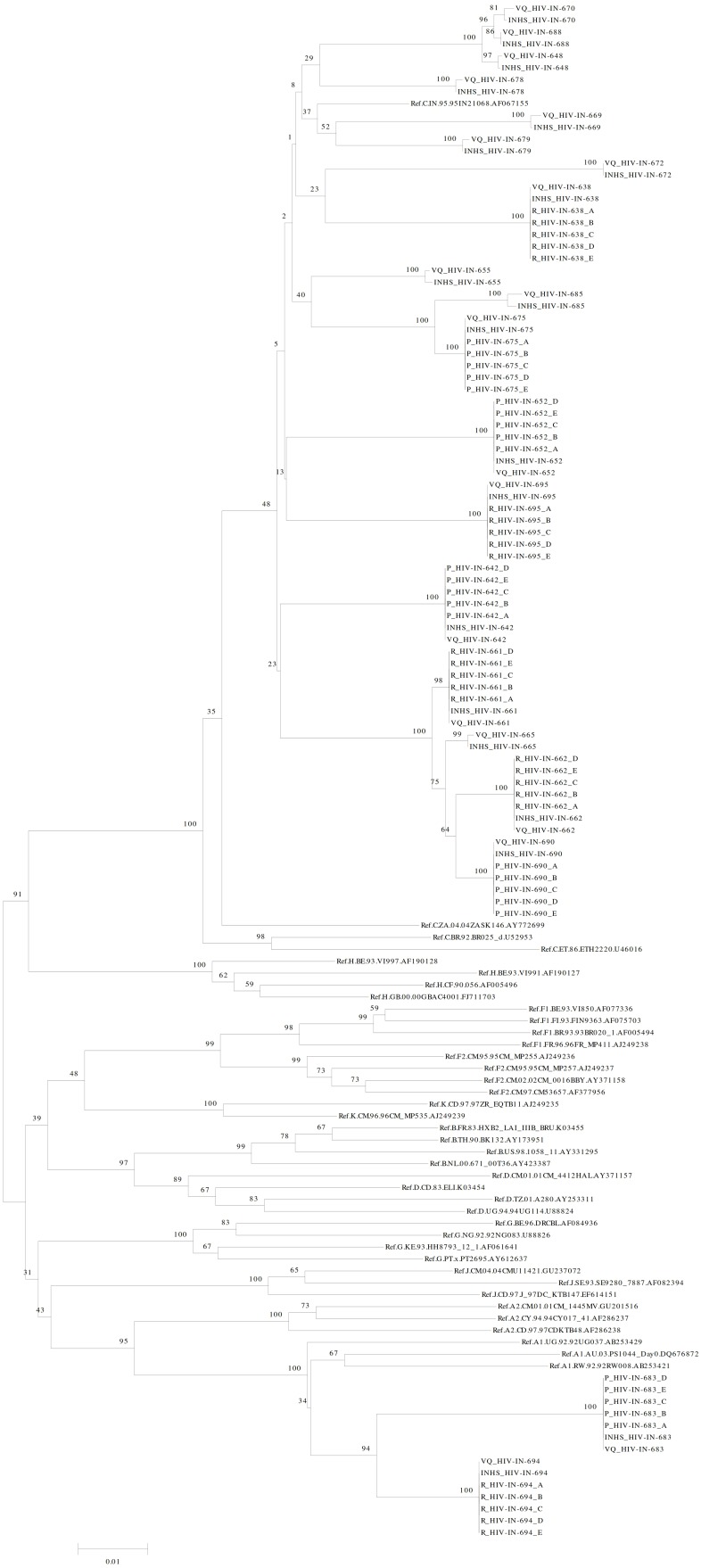
Phylogenetic Tree of reference panel samples. Phylogenetic analysis of reference panel samples showing exact correlation between in-house assay and the ViroSeq genotyping system. The construction of phylogenetic tree is described in the text. All the HIV-1 subtype reference sequences used to construct the tree were obtained from Los Alamos National Laboratory HIV sequence database (http://www.hiv.lanl.gov/content/index). VQ_ Sample ID: Sequences generated by ViroSeq assay; INHS_ Sample ID: Sequences generated by in-house genotyping assay; R_Sample ID_A to E represent reproducibility study panel while P_Sample ID_A to E represent precision study panel. The HIV-1 subtype reference sequence IDs shown in the tree are in the following order: subtype.country of origin.isolate number.accession number.

The maximum likelihood tree constructed using Mega 5.1 confirmed absence of any sample mix-up or cross contamination and sequences generated from the same sample clustered together (**Figure 1**).

### Clinical Panel result

After development of the in-house assay it was used for testing a clinical panel specially created for this purpose. The viral load and CD4+ T cell count of clinical panel samples are described in **Table 2**. Out of the 225 samples, 210 responded successfully to PCR amplification out of which 206 could be successfully sequenced and analyzed for HIV-1 drug resistance mutations. Among 110 samples with viral load of 10^5^ HIV-1 RNA copies/ml of plasma and above, 107 could be successfully amplified and both the DNA strands sequenced. On the other hand, out of 90 samples with viral load in between 10^4^ and 10^5^ HIV-1 RNA copies/ml of plasma, 82 could be successfully amplified but only 80 among them could be sequenced for both the strands. Among 13 samples with viral load in between 5×10^3^ and 10^4^ HIV-1 RNA copies/ml of plasma, 12 could be successfully amplified but double strand DNA sequence could be generated for 11 of them. Among 12 samples with viral load between 10^3^ and 5×10^3^ HIV-1 RNA copies/ml of plasma, 9 could be successfully amplified and double strand DNA sequence could be generated from 8 of them.

**Figure 2:**
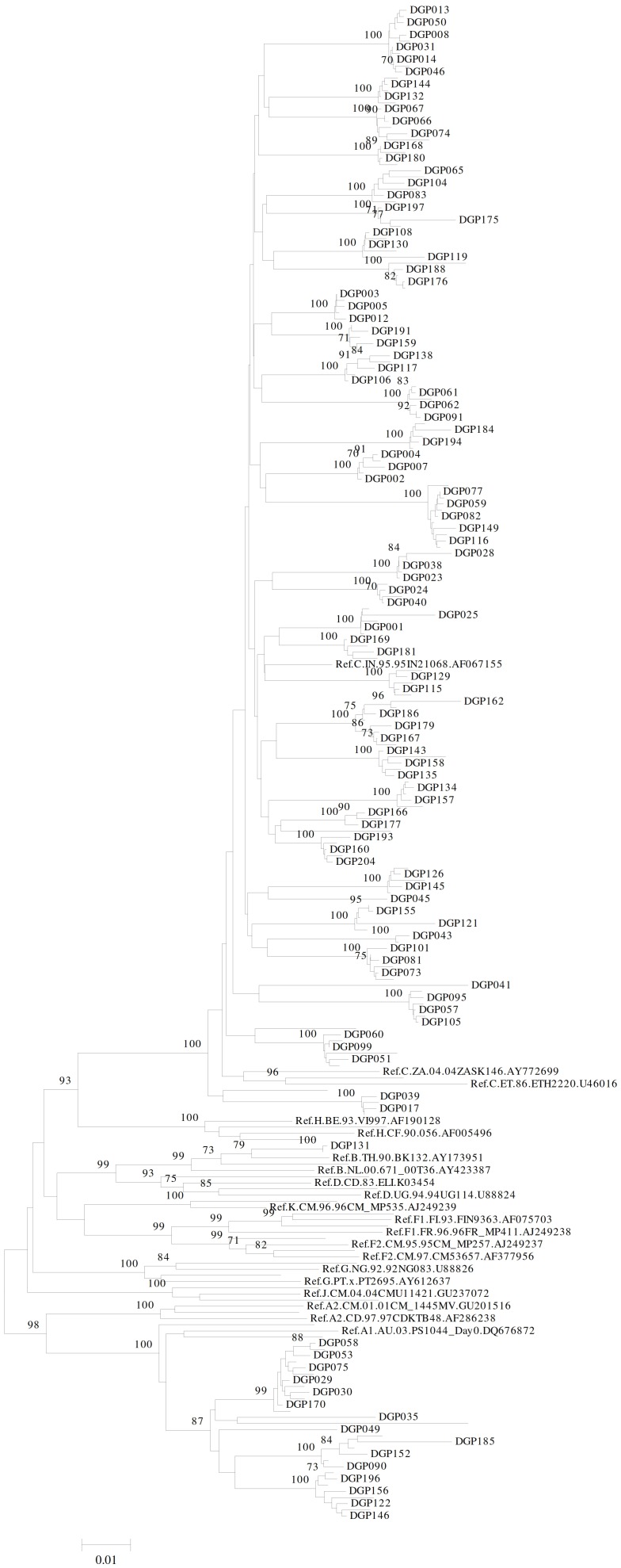
Phylogenetic tree of clinical panel samples. Phylogenetic analysis of sequences obtained from clinical panel samples. The construction of phylogenetic tree is described in the text. All HIV-1 subtype reference sequences used to construct the tree were obtained from Los Alamos National Laboratory HIV sequence database (http://www.hiv.lanl.gov/content/index). The reference sequence IDs shown in the tree are in the following sequence: subtype.country of origin.isolate number.accession number.

Out of 206 samples genotyped, 176 (85.5%), 29 (14.0%) and 1 (0.5%) were from patients infected with HIV-1 subtype C, subtype A and subtype B respectively (**Figure 2**). Samples from 28 (13.59%) patients did not show any mutations related to HIV-1 drug resistance and 178 (86.41%) of them had at least one HIV-1 drug resistance mutation(s). One hundred and fifty nine (77.18%) samples had at least one NRTI resistance mutation while 161 (78.16%) harbored at least one NNRTI mutation. Samples from 41 (19.90%) patients had at least one PI mutation(s).

**Figure 3:**
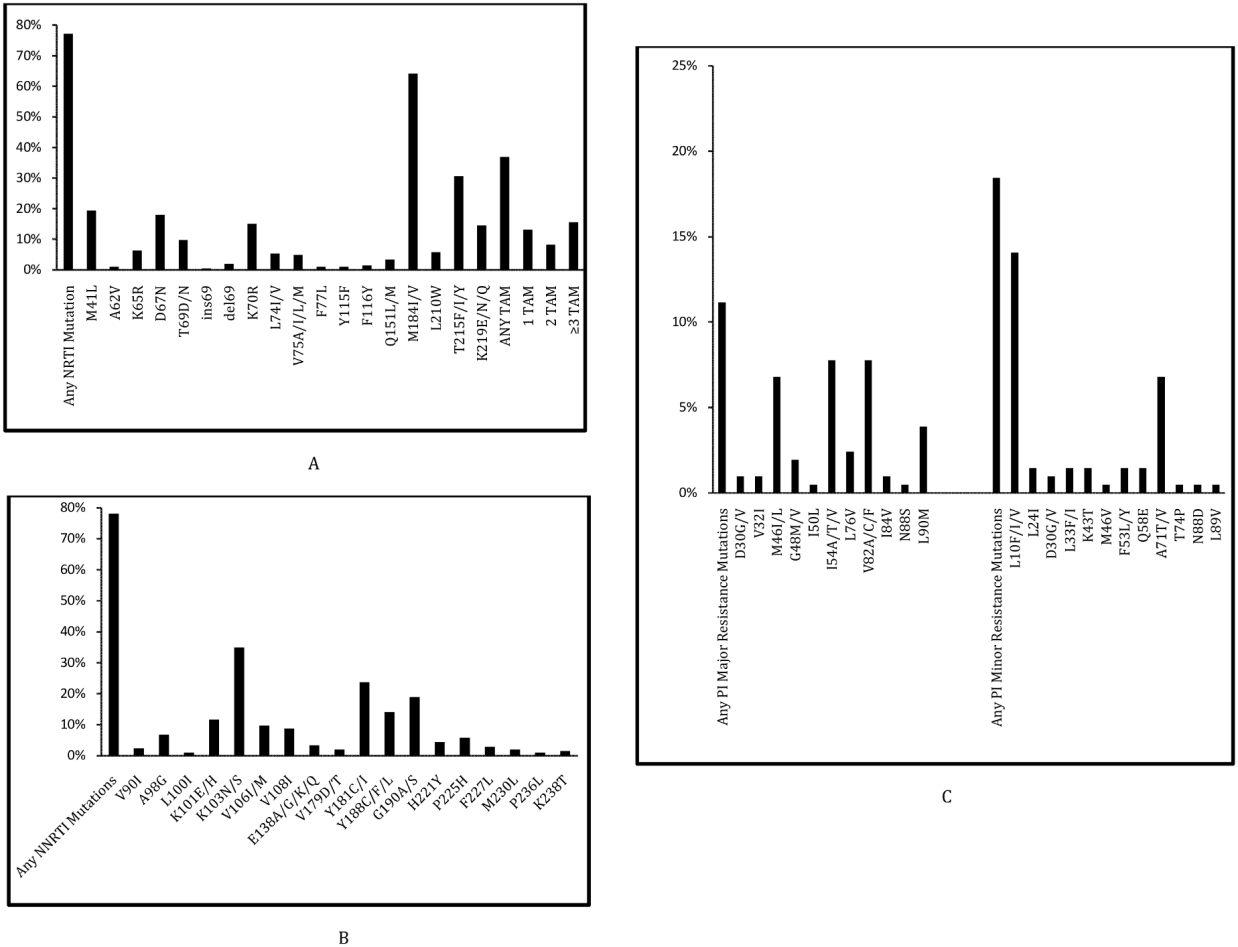
NRTI, NNRTI and PI Mutations. Frequency of Nucleoside Reverse Transcriptase Inhibitor-related drug resistance mutations [A], Frequency of Non Nucleoside Reverse Transcriptase Inhibitors-related drug resistance mutations [B] and Frequency of Protease Inhibitors-related drug resistance mutations (including PI major and PI minor drug resistance mutations) [C] in 206 patients successfully genotyped from the clinical panel failing 1^st^ line antiretroviral therapy.

All three classes of mutations were detected in samples from 29 (14.08%) patients. Among 147 patients on NNRTI and NRTI-based 1st line ART regimen, 116 had mutations belonging to these 2 categories of drugs. M184V was the most common NRTI mutation detected in 132 (64.08%) patients. 76 (36.89%) of the patients harbored at least one Thymidine Analog Mutations (TAMs). The distribution of TAMs in the clinical panel samples were M41L-40 (19.42%), D67N-37 (17.96%), K70R-31 (15.05%), L210W-12 (5.83%), T215F/Y-60 (29.12%) and K219E- 8 (3.88%) respectively. Three or more TAMs were detected in 32 (15.53%) samples while Q151M complex (Q151M, V75I, F77L and F116Y) was observed only in 2 (0.97%) patients. The details of other NRTI related mutations detected in this study are described in **Figure 3A**.

K103N was the most common NNRTI mutation present in 72 (34.95%) patients. The prevalence of other common NNRTI mutations in the study population was K101E/H-24 (11.65%), V106M-20 (9.71%), Y181C/I-49 (23.79%) and G190A/S-39 (18.93%) respectively. The details of other NNRTI-related mutations are described in **Figure 3B**.

At least one PI major or minor resistance mutations were detected in 23 and 38 patients respectively among the 59 successfully genotyped, who were exposed to ritonavir-boosted PI based 1^st^ line of antiretroviral therapy. Out of this, 20 samples harbored both PI major and minor mutations while 18 and 3 had only PI minor and only PI major mutations respectively. Eighteen samples did not have any PI-related mutations. The most common major PI mutations detected were M46I/L-14 (6.80%), I54A/T/V-16 (7.77%), V82A/C/F-16 (7.77%) and L90M-8 (3.88%) respectively. L10F/I/V-29 (14.08%) and A71T/V-14 (6.80%) were the two common PI minor mutations observed in the clinical panel. The details of other PI-related mutations are described in **Figure 3C**.

### Comparative analysis of cost and hands-on time of in-house assay with ViroSeq genotyping system

**Table 6** describes the hands-on time and cost of different stages of the analysis starting from collection of clinical sample to interpretation of drug resistance mutations. All costs are presented in US dollars. Cost of establishing the reference laboratory capable of performing the assay is however not included in the analysis. Further, some major costs common to both the assays such as logistics and manpower were also excluded. The hands-on times for the ViroSeq genotyping system and the in-house method were 18 hour 45 min and 17 hour 15 min respectively while the running cost of the in-house assay was computed at $85 compared to $303 for the ViroSeq genotyping system.

**Table 6:** Hands - on time and cost comparison between ViroSeq and the in-house HIV-1 genotyping assay.

Process	Step(s)	Assay type			
		ViroSeq		In-house	
		Hands-ontime	cost/test($)	Hands-ontime	cost/test($)
Samplepreparation	Sample collectionand plasma separation	45 min	1.0	45 min	1.0
Nucleic acidextraction	RNA Extraction	3 h	250	1 h	7.0
Amplification	One step RT-PCR	5 h 30 min		3 h 30 min	15.0
	Nested PCR			2 h 30 min	5.0
GelDocumentation	Agarose geleletrophosresis	45 min	2.0	45 min	2.0
Genotyping	Ampliconpurification	1 h		1 h	5.0
	Sequencing	2 h 30 min	30.0	2 h 30 min	30.0
	Sequence ampliconpurification	1 h 30 min	10.0	1 h 30 min	10.0
	Sequencing samplerun	2 h 30 min	10.0	2 h 30 min	10.0
Sequenceanalysis	Sequence datavalidation	30 min		30 min	
	Sequence assembly	15 min		15 min	
	Interpretation andquality analysis	30 min		30 min	
Total		18 h 45 min	303.0	17 h 15 min	85.0

## Discussion

The HIV-1 drug resistance genotyping assay is not feasible for routine monitoring of patients taking 1^st^ line antiretroviral drugs in resource limited settings like India mainly due to high cost of commercial HIV-1 genotyping assays presently available in the market [26]. But increased access to antiretroviral drugs without proper monitoring results in transmission of drug resistant HIV-1 strains in newly infected individuals [27]. Laboratory methods to monitor the treatment outcome and proper guidelines regarding course of action in case of therapeutic failure is critical in management of HIV-1/AIDS. HIV-1 drug resistance genotyping assay for patients with virologic failure acts as a guiding tool during switching to next line of treatment [12]. We performed a cost analysis of the drug resistance genotyping assay described in this study and compared it with the running cost of ViroSeq genotyping system which indicated that our assay is around 71.9% cost effective compared to the later. This attribute make this assay more suitable for routine monitoring of transmitted HIV-1 drug resistance strains as well as for detection of drug resistance mutations in patients with virologic failure.

The drug resistance mutations detected by our in-house assay exhibited excellent concordance when compared with corresponding results from the ViroSeq genotyping system. The assay was able to detect all clinically relevant mutations according to the IAS 2013 mutation list [22]. These findings demonstrate both utility and feasibility of this home brew assay in HIV-1 drug resistance surveillance and monitoring in resource limited settings like India.

None of the HIV-1 drug resistance genotyping assays including the US-FDA approved commercial assays as well as various home brew assays can successfully amplify 100% clinical samples mainly due to high genetic variability of HIV-1 [28] and occurrence of spontaneous mutation within primer binding regions of the viral genome [29]. In this backdrop, the home brew assay described in this study could successfully genotype 91% of samples from the clinical panel which was found to be satisfactory. This high rate of success is possibly due to the geographical region-specific primers designed for this assay coupled with incorporation of a nested PCR protocol.

The in-house assay described in this study was validated as per WHO guidelines for HIV-1 drug resistance genotyping and demonstrated a high degree of precision and reproducibility. The limit of detection of this assay was 1000 HIV-1 RNA copies/ml of plasma sample. This is in line with similar studies from India and other parts of the world [30]–[31]. The assay has ability to detect all major HIV-1 subtypes (HIV-1 subtype A, B and C) predominant in India [32] as revealed from the clinical panel genotyping results.

The main limitation of this study is the lack of subtype diversity in the reference panel. An ideal panel should comprise of all HIV-1 group M subtypes including circular recombinant forms which were not included here due to scarcity of such samples among HIV-1 sero-positive individuals in India. In spite of this limitation, due to the rigorous validation of assay parameters as per WHO guidelines there is enhanced confidence and reliability seen to be associated with our assay. Genotyping of the clinical panel in this study simulated real time field conditions and demonstrated good performance in detecting all clinically relevant HIV-1 drug resistance mutations in the protease and reverse transcriptase genes. This result is also in line with observations made from other similar studies reporting patterns of HIV-1 drug resistance mutations in patients failing 1^st^ file ART from India [33]–[37].

In conclusion, we report development and validation of a low cost HIV-1 drug resistance genotyping assay for resource limited settings like India with potential to serve the increasing demand of HIV-1 genotyping in the HAART *era* for effectively treating HIV/AIDS patients.
